# Mapping the global landscape of algae-based food research: A bibliometric analysis of trends, collaborations, and future directions

**DOI:** 10.1016/j.mex.2026.103923

**Published:** 2026-04-21

**Authors:** Ream N. Kinawy

**Affiliations:** College of Business Administration, Gulf University for Science and Technology, GUST Center for Sustainable Development (CSD), P.O. Box 7207, Hawally 32093, Kuwait

**Keywords:** Algae, Foods, Product development, Bibliometric analysis, Keyword co-occurrence

## Abstract

This study provides a comprehensive mapping of the global research landscape on algae, microalgae, and seaweed in food-related applications over the period 1989–2024, with particular emphasis on food additives, nutraceuticals, functional foods, and bioactive compounds. Based on 929 records retrieved from the Web of Science Core Collection, a tailored bibliometric framework is employed that integrates performance analysis, science mapping, thematic evolution, and reference-year analysis, thereby linking publication dynamics with the intellectual and conceptual structure of the field. The findings indicate a pronounced and sustained growth in algae-based food research, characterized by extensive international collaboration and a rising emphasis on functional ingredients and health-promoting compounds. Thematic and conceptual analyses delineate algal extracts, bioactive compounds, polysaccharides, proteins, and lipids as the principal knowledge clusters. The evolutionary trajectory of the field reveals a progression from primarily biochemical characterization toward more application-oriented research in food, nutraceutical, and functional health domains. Collectively, these results provide a systematic evidence base to support researchers, industry stakeholders, and policymakers in understanding current research priorities and in identifying emerging directions in algae-based food innovation.

## Graphical abstract

showcasing the exponential rise in algae-based food research (13.42 pub/year) and its high scholarly impact (37.16 citations/doc). The study highlights a 30.25 % international collaboration rate and a thematic transition from laboratory characterization to functional food applications, noting carrageenan’s 84.6 % market share alongside growing product diversification.

**Figure:**
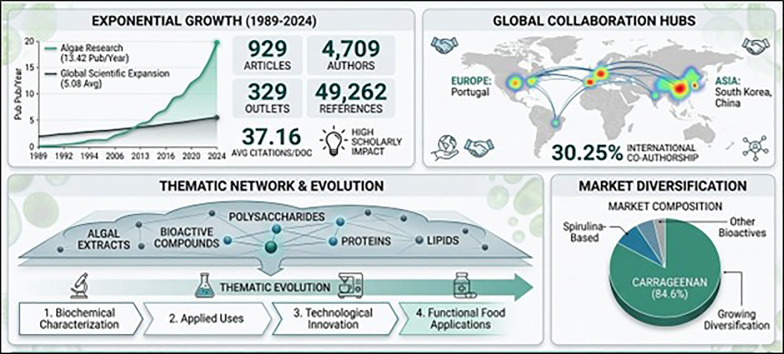

**Specifications Table**

**Table:** 

Item	Description
Subject	Food science / Marine biotechnology / Scientometrics (bibliometric analysis).
Specific subject area	Global research on algae, microalgae, and seaweed in food-related applications (food additives, nutraceuticals, functional foods; bioactive compounds such as polysaccharides, proteins, and lipids), mapped using bibliometric and network methods.
Type of data	Tables, figures, graphs; processed/filtered/analyzed bibliometric data and network visualizations (e.g., annual production trends, source/author analyses, thematic mapping).
Data collection	Records were retrieved from the Clarivate Analytics Web of Science (WoS) database using a topic-based query covering title/abstract/keywords, limited to 1989–2024.
Data processing / analysis	Bibliometric analysis performed in R (version 4.4.1) using *bibliometrix*; network visualization performed using VOSviewer (per van Eck & Waltman).
Data source location	Web of Science (Clarivate Analytics) online database; analysis conducted on exported WoS records.
Data accessibility	Data collected is available online with this version and can be collected from the WoS database using the search query: (Algae OR Microalgae OR Seaweed) AND (Food additive OR Nutraceutical OR Food supplement OR Dietary supplement OR Functional food OR Food ingredient OR Food fortification) AND (Bioactive compounds OR Polysaccharides OR Proteins OR Lipids OR Algal extract) NOT (Biofuel OR Energy); timespan 1989–2024.

**Value of the Data**

❖ These data quantify long-term publication growth and knowledge structure for algae-based food research (1989–2024), enabling evidence-based understanding of how the field has evolved.
❖ The collaboration and productivity indicators (e.g., authorship patterns and international co-authorship) help researchers and institutions identify key hubs and potential collaborators.
❖ The keyword, thematic, and conceptual mapping highlights dominant and emerging topics (e.g., algal extracts, bioactive compounds, polysaccharides; functional foods/nutraceuticals), supporting hypothesis generation and research prioritization.
❖ The results can inform industry and policy stakeholders by clarifying where research attention concentrates (themes, sources, institutions) and where opportunities for innovation remain.

## Background

Global food security is currently experiencing unprecedented pressure, as population growth outpaces the typical rates of conventional agricultural production [1]. This imbalance underscores the urgent necessity to identify and develop innovative, sustainable, and alternative protein sources capable of meeting increasing nutritional demands [2]. Amid these challenges, algae, including both microalgae and macroalgae, have attracted substantial scientific and industrial interest as alternative food additives and nutritional supplements and are now positioned at the nexus of sustainability, human nutrition, and biotechnology [3,4]. This upward trend is evidenced by the expanding market for algae-derived products, which is projected to increase in value from USD 5.87 billion by 2025 to USD 8.07 billion by 2030. This market growth is driven primarily by rising consumer demand for sustainable, protein-based products and by heightened awareness of the health benefits associated with algal omega-3 fatty acids and other bioactive compounds [4,5].

Algae have a unique proportionality of nutritional values that are highly beneficial and differentiate them among the standard sources of food [6]. These organisms store proteins which are up to 70 % of their dry weight, and this has made them become outstanding alternative sources of protein to be used by humans and in animal feeds as well [7,8]. In addition to protein, algae are found to be a source of bioactive compounds such as polysaccharides, such as alginate, fucoidan, carrageenan, and agar; phycobiliproteins; carotenoids like astaxanthin and beta-carotene; phenolic compounds; vitamins; and minerals [9]. The bioavailability and efficacy of these ingredients have been reported throughout a large body of scientific research and have shown effects on metabolic indicators of body health, such as glucose homeostasis, lipid profiles, and inflammatory markers [10,11]. Moreover, the environmental benefits of algae cultivation, such as low freshwater needs, the possibility to grow in arid and saline-contaminated lands that cannot support conventional food production, and the possibility of carbon dioxide capture at the same time, make algal biomass an ecologically compatible food production system [12,13]. The range of uses of algae-derived products has grown far beyond simple supplementation. The modern food industry applications include the use of algae in functional foods, nutraceutical preparations, bakery, beverages, dairy preparation, and fortified snacks [14]. Algae-derived components that are commercially available and approved as food and feed additives include astaxanthin, β-carotene, phycocyanin, fatty acids found in algae, and whole algal biomass products (*Spirulina* and *Chlorella*), and it is being demonstrated that many more algal species and bioactive compounds could have potential applications [15,16]. This broadening scope of application is indicative not only of technological innovations in the extraction and processing technique, but also a body of accumulating clinical evidence to support the promotion of health [17].

Along with the development of algae use in food systems, the level of scientific research in this area has grown exponentially. Bibliometric analysis has become a performance that cannot be done without a synthesis of scientific literature in large amounts and the determination of new trends, the network of collaboration, and an assessment of the intellectual organization of the research [18]. Bibliometric methods use mathematical and technical ways of quantitative analysis of published literature to allow a researcher to identify trends in scientific output, recognize seminal contributions, map thematic links, and predict future research trajectories [19]. Such systematic bibliometric reviews provide the research community with a comprehensive overview of the scientific landscape, thereby facilitating evidence-based identification of research gaps, emerging opportunities, and potential avenues for collaboration. To date, although individual reviews have examined specific aspects of algae utilization in food applications such as nutritional properties, functional characteristics, and technological or processing uses, there remains an absence of an in-depth bibliometric synthesis of the broader body of research. In particular, no existing work has systematically integrated analysis of the publication corpus, key contributing authors and institutions, developments in educational curricula, thematic evolution, and prospective future research directions. The present study addresses this knowledge gap by employing a rigorous bibliometric methodology that systematically interrogates the published literature to generate quantitative insights into research trajectories, productivity patterns, and prevailing conceptual frameworks related to the use of algae within food systems. This comprehensive assessment is intended to provide researchers, industry stakeholders, and policy-makers with a robust, evidence-based understanding of both the current state and emerging priorities in the field. In doing so, it aims to support informed strategic decision-making and to foster the development of interdisciplinary collaborations in an area of inquiry that is currently experiencing rapid, exponential growth.

## Research methodology

### Research design and analytical approach

**Fig. 1:**
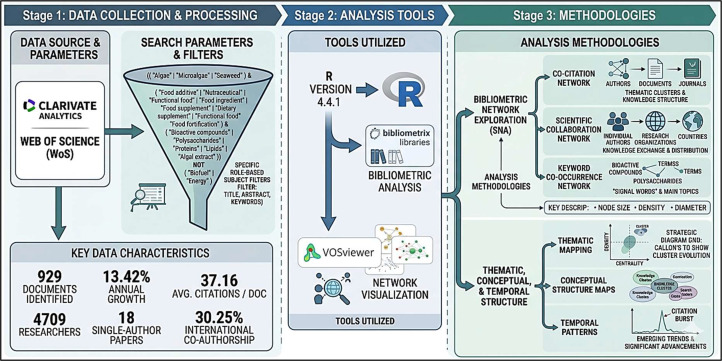
Schematic depiction of the bibliometric study design and associated analytical framework.

This study employed a bibliometric approach to systematically map and evaluate the scientific literature on algae and their applications as food additives. Bibliometric analysis was selected because it enables the quantitative examination of publication dynamics, citation impact, collaboration networks, conceptual development, and the intellectual structure of a delineated research field. The methodological workflow comprised three sequential stages: (1) data collection and processing, including database selection, formulation of the search strategy, data retrieval, eligibility screening, and subsequent cleaning and standardization of bibliographic records; (2) application of analytical tools for both descriptive and network-based bibliometric analyses; and (3) methodological interpretation via thematic, conceptual, and temporal analyses of the retrieved literature, as illustrated in (Fig. 1). Furthermore, this investigation incorporated a customized bibliometric design by integrating performance analysis, science mapping, thematic evolution analysis, and reference-year analysis into a single analytical framework. In contrast to studies that report only publication counts or keyword frequencies, the present approach establishes explicit linkages between productivity patterns and the underlying knowledge structure, conceptual trajectories, and historical foundations of the field. This integrated design was intended to yield a more comprehensive understanding of the evolution of research on algae-based food additives over time and to identify the key actors, thematic areas, and seminal documents that have shaped this domain. The methodological novelty of this study resides in the customization of bibliometric procedures to the specific domain of algae-based food research. Unlike generic bibliometric studies that rely primarily on publication counts, citation indicators, or isolated keyword mapping, the present study integrates performance analysis, co-citation and collaboration mapping, keyword co-occurrence analysis, thematic structure analysis, and cited-reference year analysis into a single analytical framework. This design enables simultaneous examination of productivity patterns, intellectual structure, conceptual evolution, and historical foundations of the field.

### Data source and search scope

The bibliographic dataset was retrieved from the Web of Science (WoS) Core Collection, a multidisciplinary citation index curated by Clarivate Analytics. WoS was selected as the data source due to its broad disciplinary coverage, the high degree of standardization and structuring of its bibliographic records, and its widespread adoption in bibliometric and scientometric studies, particularly those relying on citation-based analyses [20,21]. Only documents indexed in WoS that met the predefined search criteria were included in the analysis. The temporal coverage of the search extended from 1989 to 2024. The final search was executed on this database, and all records available within WoS that conformed to the search parameters were considered eligible for subsequent screening. To enhance retrieval precision and ensure thematic relevance, the search strategy applied a topic-field restriction (TS), which concurrently queried the title, abstract, author keywords, and Keywords Plus fields. The final search query was specified as follows: TS = ((“Algae” OR “Microalgae” OR “Seaweed”) AND (“Food additive” OR “Nutraceutical” OR “Food supplement” OR “Dietary supplement” OR “Functional food” OR “Food ingredient” OR “Food fortification”) AND (“Bioactive compounds” OR “Polysaccharides” OR “Proteins” OR “Lipids” OR “Algal extract”)) NOT (“Biofuel” OR “Energy”). This search strategy was explicitly constructed to optimize the retrieval of publications about the utilization of algae in food-related additives, nutraceuticals, and functional applications, while systematically excluding records whose primary emphasis concerned energy generation or biofuel production. The integrated application of Boolean operators, synonymous and semantically related terms, and exclusion criteria was designed to enhance both the breadth of coverage (recall) and the thematic specificity (precision) of the resulting dataset.

### Eligibility criteria and dataset construction

To ensure the relevance and reproducibility of the dataset, explicit inclusion and exclusion criteria were defined and systematically applied. Records were included if they: (1) focused on algae, microalgae, or seaweed; (2) addressed food additives, food ingredients, nutraceuticals, dietary supplements, functional foods, or related food fortification applications; and (3) contained terminology associated with algal bioactive compounds, including polysaccharides, proteins, lipids, or algal extracts. Records were excluded when their primary emphasis concerned biofuel production, energy-related applications, or non-food industrial uses. The screening procedure was implemented in two sequential stages. First, records were pre-filtered using the WoS search query in combination with the specified temporal restriction. Second, titles, abstracts, and author keywords were manually inspected to verify thematic pertinence and to eliminate records that were clearly outside the scope of the study. In instances where relevance could not be determined based solely on these elements, the complete record metadata was examined manually. Following this multistep screening process, the final dataset consisted of 929 documents, comprising both original research articles and review papers, which constituted the analytical corpus for the subsequent bibliometric analysis.

### Data processing and reproducibility protocol

The retrieved records were exported from WoS in BibTeX format, including complete bibliographic metadata. The resulting file was subsequently imported into R (version 4.4.1) for data preprocessing and bibliometric analysis. Data management procedures were implemented using the Bibliometrix package, and network visualizations were generated with VOSviewer. To enhance reproducibility, preprocessing followed a predefined, sequential workflow. First, the exported records were imported into R. Second, bibliographic fields were examined for completeness and internal consistency. Third, document types were screened to retain only those publication categories specified as eligible in the study protocol. Fourth, author names, keywords, and source titles were assessed for standardization, including the harmonization of evident spelling variants and plural/singular forms when they referred to the same conceptual entity. Fifth, the cleaned dataset was employed for descriptive, network, and thematic analyses. Because the dataset originated from a single bibliographic database, inter-database deduplication was not required; nonetheless, Zotero was additionally used to perform a duplicate check.

### Bibliometric performance analysis

**Fig. 2:**
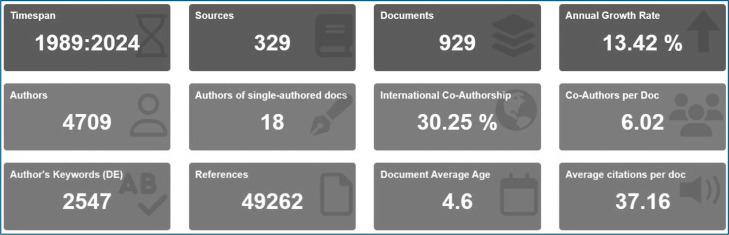
Key data characteristics of current research about the usage of algae in food additives.

Descriptive bibliometric indicators were initially computed to characterize the dataset; these indicators comprised annual scientific output, annual growth rate, mean citations per document, total number of authors, number of single-authored documents, and the rate of international co-authorship (Fig. 2). Based on the retrieved dataset, the field encompassed 929 publications, exhibited an annual growth rate of 13.42 %, involved 4709 distinct authors, and showed an average of 37.16 citations per document. Moreover, 18 documents were single-authored, and the international co-authorship rate reached 30.25 %. A performance analysis was subsequently conducted to identify the most productive publication years, the most influential sources, the leading authors, and the prevailing citation patterns within the field. This stage established the empirical baseline for the subsequent science-mapping analyses by elucidating the scale, temporal growth dynamics, and productivity structure of research on algae-based food additive applications.

### Network analysis and parameter settings

A science-mapping analysis was conducted to delineate the intellectual, social, and conceptual structures of the research domain. Three principal types of networks were examined: co-citation networks, collaboration networks, and keyword co-occurrence networks. In these networks, nodes represented analytical units such as authors, documents, journals, countries, institutions, or keywords, whereas edges encoded the relationships among these units, including co-citation frequency, co-authorship ties, or the strength of keyword co-occurrence. Co-citation analysis was applied to identify seminal references, influential authors, and core knowledge clusters that constitute and shape the cognitive backbone of the field. Collaboration analysis was undertaken at the levels of authors, institutions, and countries to characterize the structure, intensity, and patterns of scientific cooperation. Keyword co-occurrence analysis was used to detect dominant research themes, emerging topical areas, and conceptual linkages within the scholarly corpus. To ensure methodological transparency and reproducibility, the construction of bibliometric networks was guided by explicitly specified analytical parameters. A full-counting scheme was adopted for all analyses, thereby preserving the complete contribution of each bibliographic record to the overall network structure. The units of analysis varied according to the network type under consideration and encompassed authors, institutions, countries, cited documents, cited journals, and author keywords. Threshold values were determined a priori to balance inclusiveness and interpretability and to avoid excessively fragmented or noisy visual representations. Accordingly, only nodes that satisfied the minimum required number of publications, citations, or keyword occurrences were retained for mapping, whereas items falling below these thresholds were excluded from the final network visualizations.

### Thematic, conceptual, and temporal analysis

To complement the network analysis, thematic and conceptual structure analyses were undertaken to elucidate the organizational patterns and temporal evolution of the research domain. Thematic mapping was employed to classify research topics according to their centrality and density, thereby differentiating motor themes, basic themes, niche themes, and emerging or declining themes. This procedure facilitated the identification of topics that are both highly developed and central to the field, as well as those that remain peripheral or are only beginning to emerge. Conceptual structure analysis was additionally applied to detect broader knowledge clusters and to characterize thematic relationships among terms within the corpus. Furthermore, the historical interpretation of the field was informed by examining the publication years of cited references, which offered insight into the temporal foundations of the domain and the trajectory of its intellectual development. Collectively, these analytical approaches enabled the study to move beyond simple productivity measures and to explicate how research on algae-related food additives has evolved conceptually over time.

### Software environment

All statistical and bibliometric analyses were performed in R (version 4.4.1) using the Bibliometrix package. R was selected because it offers a transparent and reproducible computational environment for the import, preprocessing, analysis, and visualization of bibliographic data. In addition, VOSviewer was employed to construct and examine bibliometric network visualizations, owing to its demonstrated strengths in mapping co-authorship, co-citation, and keyword co-occurrence structures. The joint application of Bibliometrix and VOSviewer enabled systematic cross-validation between quantitative analytical outputs and corresponding visual network representations. This software configuration also constitutes a methodological refinement of the present study, whereby Bibliometrix was used for reproducible statistical analysis and thematic exploration, while VOSviewer was dedicated to the high-resolution visual mapping of network relationships.

### Technical validation of the method

Technical validation was conducted to evaluate the reliability of dataset construction and the robustness of the derived bibliometric structures. Retrieval validity was established through manual verification of the thematic relevance of records obtained via the WoS search strategy. Preprocessing validity was reinforced by systematic metadata standardization and duplicate detection, implemented using Bibliometrix-supported processing workflows in combination with Zotero-assisted screening procedures. Analytical validity was further assessed by comparing the principal bibliometric patterns generated independently by Bibliometrix and VOSviewer, thereby providing cross-platform corroboration of the main collaboration networks, citation structures, and thematic clusters identified in the dataset.

## Results

The study of algae, microalgae, and seaweed as food additives, nutraceuticals, and sources of bioactive substances has been a subject of continuous and increasing attention from 1989 to 2024, as clear in (Table 1). The study of 929 papers has been enriched by a total of 329 sources. These documents, with an average age of 4.6 years, demonstrate the research's up-to-date nature and its ongoing development. The yearly growth rate of 13.42 % emphasizes the increasing progress and significance of this field, with an average of 37.16 citations per document demonstrating its influence. The dataset, consisting of 49,262 references, demonstrates the substantial research endeavors undertaken. The presence of various document formats, such as articles, reviews, and book chapters, along with the utilization of 2644 distinct identifiers and 2547 separate keywords, indicates a broad spectrum of topics being investigated. The research findings indicate a substantial level of collaboration, with the involvement of 4709 authors and international collaborations in 30.25 % of the documents. This suggests a strong global interest and cooperation in the study. This data highlights the potential of algae and related organisms to contribute to sustainable and health-promoting food solutions. The continual expansion and collaboration in this field offer great opportunities for further discoveries and applications.

**Table 1:** Presents key data on the study on the application of algae in food additives.

Description	Results
MAIN INFORMATION ABOUT DATA	
Timespan	1989:2024
Sources (Journals, Books, etc.)	329
Documents	929
Annual Growth Rate%	13.42
Document Average Age	4.6
Average citations per doc	37.16
References	49262
DOCUMENT CONTENTS	
Keywords Plus (ID)	2644
Author's Keywords (DE)	2547
AUTHORS	
Authors	4709
Authors of single-authored docs	18
AUTHORS COLLABORATION	
Single-authored docs	19
Co-Authors per Doc	6.02
International co-authorships%	30.25
DOCUMENT TYPES	
Article	618
article; book chapter	29
article; early access	9
article; proceedings paper	15
editorial material	1
proceedings paper	8
Review	236
review; book chapter	4
review; early access	9

### Annual scientific production

**Fig. 3:**
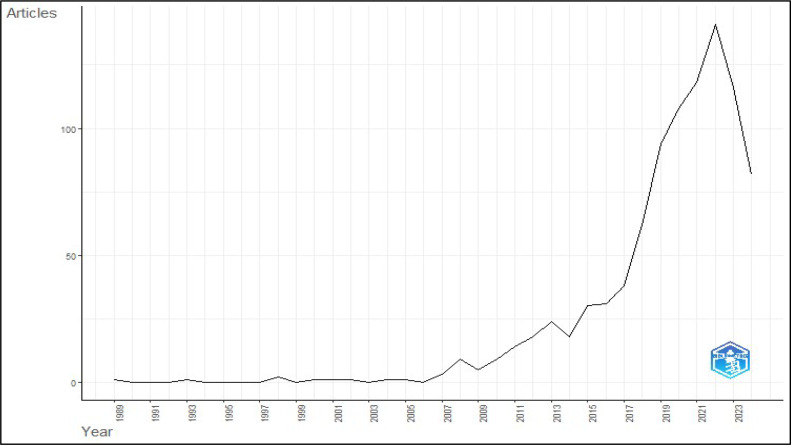
Annual scientific production of research on the usage of algae in food additives.

The yearly scientific output statistics on research on algae in the context of food additives (Fig. 3), nutraceuticals, and bioactive substances show a noticeable increasing trend, especially starting from 2007. From 1989 to 1999, there was a lack of scientific effort, characterized by infrequent publications. The period from 2000 to 2006 maintained the pattern of poor production; however, a significant rise commenced in 2007. Between 2007 and 2011, there was a gradual increase in scientific output, reaching a peak of 14 papers by 2011. There was a notable increase in the number of articles from 2012 to 2017, with the count rising from 18 to 38. The period from 2018 to 2022 had a significant rise in publications, reaching its highest point in 2022 with 141 articles. Nevertheless, there was a modest decrease in the number of articles in 2023 and 2024, with 116 and 82 articles, respectively. The upward trajectory in scientific production indicates a rising enthusiasm and financial commitment towards studying algae and their potential uses in food additives and bioactive chemicals, particularly starting from 2007. The surge in 2022 is probably a result of a confluence of heightened research endeavors, possibly propelled by notable advancements or augmented financial support. The marginal decrease observed in 2023 and 2024 could potentially be ascribed to alterations in research goals, modifications in funding, or the inherent variability in scientific productivity. Although there has been a decrease, the general pattern still shows a positive direction, suggesting that the study of algae is important and constantly changing, with continuous contributions to sustainable and health-enhancing food solutions. This tendency indicates that the field is becoming more developed and may be entering a stage where the emphasis moves from exploration to practical implementation and creative advancement.

**Fig. 4:**
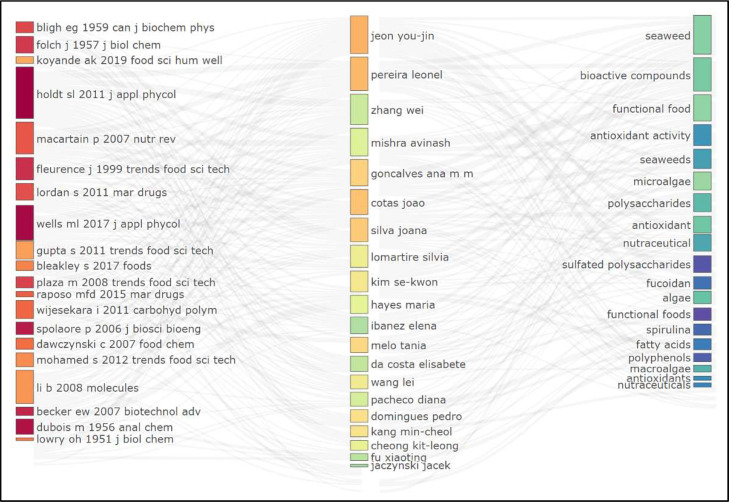
The three-fold plot analysis of research on the utilization of algae in food additives.

Furthermore, (Fig. 4) highlights three essential components: “References, Authors, and Keywords,” each offering unique insights into the study's dynamics. The study emphasizes the lasting influence of foundational works by Bligh EG (1959) and Folch J (1957), which remain pivotal in this research theme, while also recognizing the significant contributions of Holdt SL (2011) and Mecartain P (2007) in advancing the understanding and application of algae within food additives. The plot further underscores the achievements of researchers like Joon You-Jin, Pereira Leonel, and Zhang Wei, who have made substantial progress in the fields of bioactive compounds and functional foods. The diverse authorship, including figures such as Mishra Avinash, Silva Joana, and Hayes Maria, reflects broad international collaboration, underscoring the interdisciplinary nature of this research. The frequent references to terms like "bioactive substances," "functional food," "antioxidant activity," and "polysaccharides" highlight the core themes of the study, which focuses on the health benefits and functional properties of algae-derived compounds. This tripartite framework effectively illustrates the collaborative and multidisciplinary efforts in algal research, emphasizing its potential to advance health-promoting and functional food products. The numerous citations of seminal studies indicate a well-established body of knowledge that continues to influence current research, while the presence of a wide array of authors and keywords points to a dynamic and expanding field, characterized by significant international cooperation. Overall, the graphic underscores the growing importance of algae in developing innovative solutions for food and health, particularly through the exploration of bioactive compounds and their antioxidant properties, with ongoing research and expanding collaboration signaling a promising future for advancements in this field.

### Most relevant sources

**Fig. 5:**
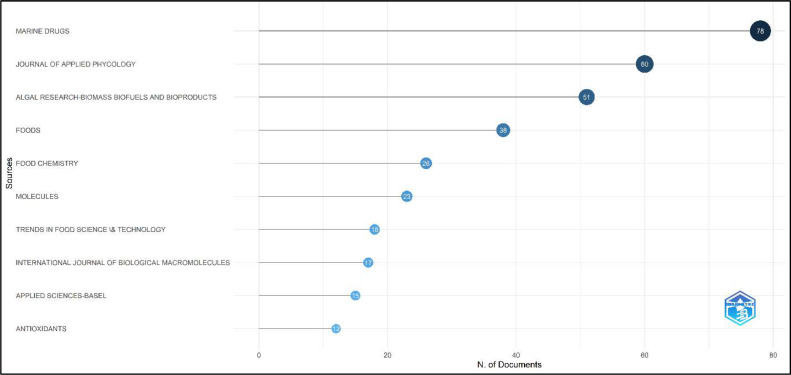
The most relevant sources of research on the utilization of algae in food additives.

An examination of the primary sources in the field of algae research pertaining to food additives, nutraceuticals, and bioactive substances identifies the prominent publications that have achieved noteworthy advancements in this area (Fig. 5). The prominent scientific publication, MARINE DRUGS, features a total of 78 articles, highlighting its significant focus on bioactive chemicals originating from marine sources and their practical utilization in the fields of food and health. The JOURNAL OF APPLIED PHYCOLOGY, containing 60 articles, specifically emphasizes the practical utilization of phycology in food technology. On the other hand, ALGAL RESEARCH-BIOMASS BIOFUELS AND BIOPRODUCTS, with 51 articles, covers a wide range of topics that go beyond biofuels, including bioactive compounds and applications related to food. Prominent scientific publications like FOODS and FOOD CHEMISTRY, with 38 and 26 articles respectively, demonstrate a specific emphasis on the chemical characteristics and practical applications of food ingredients obtained from algae. Moreover, MOLECULES and TRENDS IN FOOD SCIENCE & TECHNOLOGY, both consisting of 23 and 18 articles respectively, encompass a wide range of subjects, encompassing molecular composition and technological advancements in the field of food science. The INTERNATIONAL JOURNAL OF BIOLOGICAL MACROMOLECULES and APPLIED SCIENCES-BASEL are specialized journals that focus on algae research, covering both its biological and applied aspects. The former has published 17 articles on the subject, while the latter has published 15. ANTIOXIDANTS and CRITICAL REVIEWS IN FOOD SCIENCE AND NUTRITION are scholarly journals that focus on the health benefits and critical analysis of studies in the field of food science. Each journal contains 12 articles. The allocation of articles among these many journals demonstrates the interdisciplinary character of algal research, connecting marine biology, food science, chemistry, and applied sciences. The significant focus on bioactive chemicals and functional foods highlights the potential of algae as a crucial resource in the development of food items that improve health and provide enhanced nutrition. This extensive survey of the most often cited sources functions as a great resource for scholars and professionals, assisting in the recognition of crucial publications for more study, research submissions, and keeping abreast of the latest advancements in this ever-changing discipline.

**Fig. 6:**
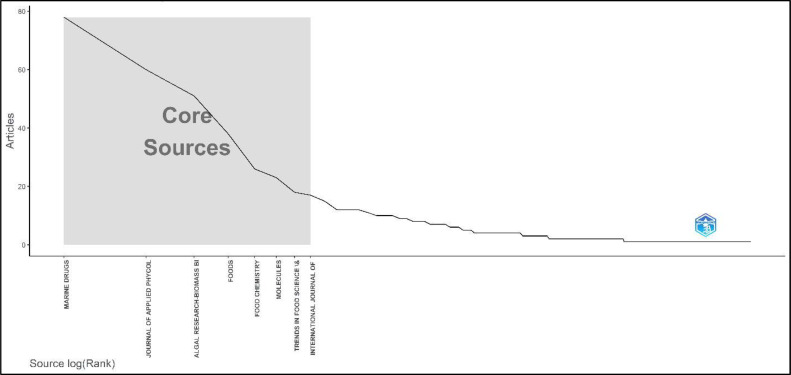
The core sources by Bradford’s Law of research on the utilization of algae in food additives.

Additionally, the use of Bradford's Law (Fig. 6) in data analysis reveals the key scientific publications that make a substantial contribution to research on algae in the field of food additives, nutraceuticals, and bioactive chemicals. Marine Drugs tops the list with 78 papers, highlighting its significance as a primary resource for studying bioactive substances originating from the sea in relation to food and health uses. The Journal of Applied Phycology, containing 60 articles, specifically emphasizes the practical utilization of phycology, particularly in the field of algae application in food technology. On the other hand, Algal Research-Biomass Biofuels and Bioproducts has published 51 papers that are pertinent to its wide-ranging scope, extending beyond its title. Additional notable contributors include Foods and Food Chemistry, which investigate the chemical and functional characteristics of food ingredients derived from algae, as well as journals such as Molecules and Trends in Food Science & Technology, which focus on the molecular composition and technological progress in the field of food science. The International Journal of Biological Macromolecules and Applied Sciences-Basel and Antioxidants and Critical Reviews in Food Science and Nutrition are specialized journals that respectively emphasize the biological and applied sciences aspects of algae research and the health benefits and critical evaluations in food science. The wide variety of publications in this field demonstrates the interdisciplinary nature of the research, highlighting the potential of algae as a useful resource for creating food items that promote health and have higher nutritional value. Bradford's Law offers a thorough analysis that helps scholars and practitioners discover important journals for further study, research submissions, and staying informed about the newest developments in the field.

**Fig. 7:**
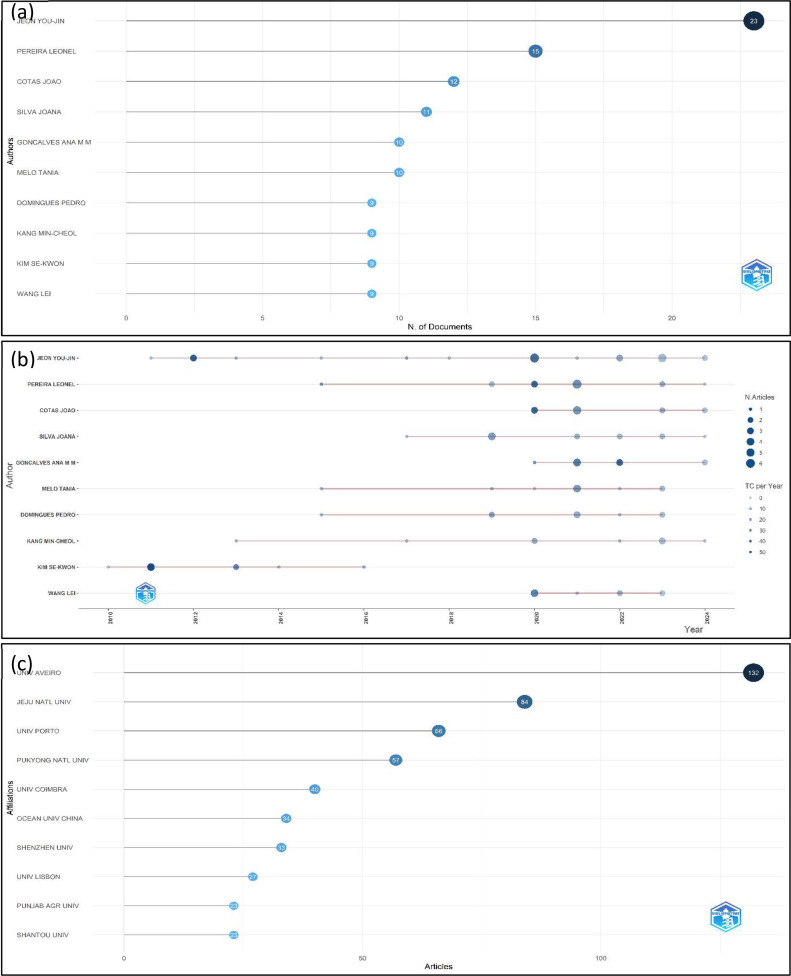
(a) The most relevant authors' research, (b) Shows Authors' Production over Time, (c) most relevant affiliations about the utilization of algae in food additives.

A comprehensive analysis of the prominent authors in the domain of algae research about food-related applications, as determined by the quantity of published publications, reveals a number of significant contributors and budding experts (Fig. 7, a). JEON YOU-JIN stands out as a prominent leader in the field, having authored 23 publications and a fractionalized count of 4.63. This highlights their substantial impact on academic literature. Following closely is PEREIRA LEONEL, who has produced 15 publications with a fractionalized count of 2.26, making a significant contribution to the advancement of the discipline. With a total of 12 papers and a fractionalized count of 2.02, COTAS JOAO has established itself as a noteworthy entity in this particular field. Further distinguished researchers are SILVA JOANA and GONCALVES ANA M. M., who have made significant contributions of 11 and 10 articles, respectively, demonstrating their active engagement in joint research endeavors. Despite having published a total of 9 papers, Kim Se-KWON has achieved a noteworthy fractionalized count of 3.87, which suggests large individual contributions. Emerging scholars, namely LOMARTIRE SILVIA and HAYES MARIA, have contributed 8 and 7 publications respectively, while ZHANG WEI and CHEONG KIT-LEONG have contributed 7 and 6 articles respectively, thereby establishing themselves as prominent figures in this particular field of research. This analysis elucidates the collaborative and interdisciplinary characteristics of algae research and its applications in the field of food science, underscoring the significant contributions made by these authors and their leadership in propelling the field forward. This information may be of significant value to researchers as it can aid in the identification of key collaborators, influential studies, and potential mentors within this subject area of study. While (Fig. 7, b) provides a thorough graphical depiction of publication patterns and the influence of different scholars in the domain of investigating algae for applications related to food. This graph illustrates the publication output of individual authors over time, with each author being depicted by a line of dots. Larger dots are indicative of a greater quantity of publications within a specific year, whilst dots that are color-coded represent the total number of citations per year (TC per Year), thus reflecting the impact of their literary contributions. The analysis of the chart indicates that authors such as JEON YOU-JIN exhibit a consistent pattern of research activity over an extended period, showcasing their enduring contributions to the associated subject. Likewise, PEREIRA LEONEL and COTAS JOAO demonstrate consistent levels of publishing output, characterized by significant surges in specific years. The chart also highlights the notable citation impact of authors such as JEON YOU-JIN and KIM SE-KWON, who are specifically identified by frequent, huge, color-coded dots, underscoring their powerful positions within the research community. LOMARTIRE SILVIA and HAYES MARIA, among other emerging researchers, have garnered recognition for their escalating publication patterns, indicating their burgeoning significance within the academic domain. The presence of overlapping publishing years among numerous authors indicates a collaborative research environment, which is a defining feature of interdisciplinary areas such as algal research. This analysis examines the relationship between productivity and citation impact, offering useful insights into the primary contributors and changing dynamics within the field. It assists researchers in discovering possible collaborators, staying updated on influential papers, and acknowledging prominent specialists. Also, the analysis of affiliations in research (Fig. 7, c), reveals significant contributions from global institutions, highlighting the international scope and collaborative nature of this field. Leading the contributions is the University of Aveiro in Portugal, with 132 publications, underscoring its strong focus on algae and bioactive compounds for food and nutraceutical applications. Jeju National University in South Korea follows with 84 publications, reflecting its emphasis on marine biotechnology and the use of seaweed and microalgae in functional foods. The University of Porto, also in Portugal, contributes 66 publications, further emphasizing Portugal's prominent role in this research area. Other key contributors include Pukyong National University in South Korea and the University of Coimbra in Portugal, with 57 and 40 publications, respectively. The data also highlights notable contributions from Asian and European institutions, including the Ocean University of China and Shenzhen University, which emphasize the importance of this research in Asia, and institutions like the University of Lisbon and University College Dublin, which showcase Europe's robust research network. Additionally, global institutions from Australia, South America, the Middle East, and Southeast Asia, such as the University of Melbourne, the University of São Paulo, Islamic Azad University, and University Putra Malaysia, demonstrate the widespread research interest in this field. The data suggests that European and Asian institutions, particularly in Portugal and South Korea, are leading advancements in algae-related studies, likely driven by the availability of rich marine resources and a strategic focus on developing functional foods and nutraceuticals. The global participation from institutions across different continents underscores the universal importance and potential of algae and microalgae in contributing to the food and nutraceutical industries, positioning these institutions as key players in driving future innovations and applications in the field.

### Documents, cited references, and words

**Fig. 8:**
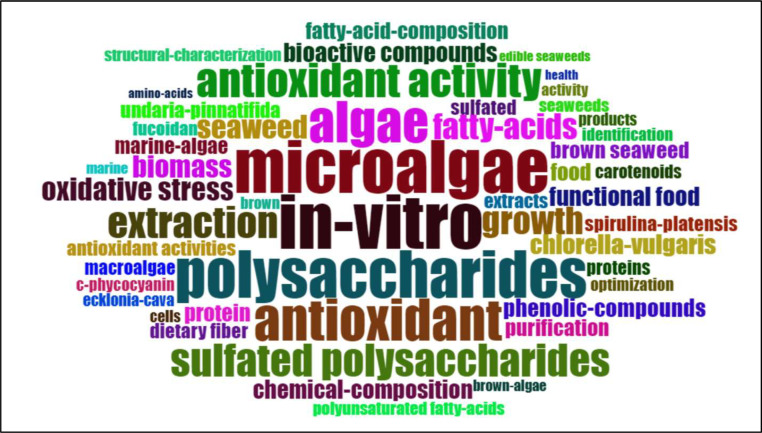
Keyword-based word cloud of the most frequent algal utilization in food research terms.

The analysis of key terms in research on algae in their applications as food additives, nutraceuticals, dietary supplements, functional foods, and bioactive compounds (Fig. 8) reveals that "in-vitro" is the most frequently occurring term, appearing 131 times. It is followed by "microalgae" (127 occurrences), "polysaccharides" (114 occurrences), and "antioxidant" (101 occurrences). The prominence of "in-vitro," "microalgae," and "polysaccharides" reflects a substantial focus on laboratory-based studies and the investigation of specific bioactive compounds from microalgae. The frequent references to "antioxidant" and "antioxidant activity" (83 occurrences) indicate a significant interest in the health benefits of these compounds, especially their potential to mitigate oxidative stress. Additionally, terms such as "extraction," "growth," and "biomass" highlight ongoing research aimed at optimizing the production and extraction processes of these valuable compounds. Overall, this word frequency analysis emphasizes the considerable research efforts devoted to understanding and leveraging the health-promoting properties of algae-derived substances for various food and nutraceutical applications.

**Fig. 9:**
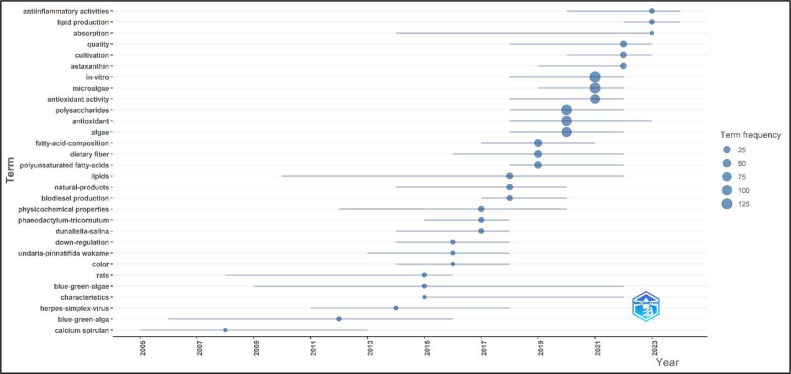
Algal utilization in food research trending topic over time.

The analysis of trending topics (Fig. 9) shows that terms such as “anti-inflammatory activities,” “lipid production,” “cultivation,” and “astaxanthin” have shown significant frequency over recent years. The prominence of “anti-inflammatory activities” indicates a strong research focus on the health benefits of algae-derived compounds, particularly their potential to reduce inflammation. “Lipid production” and “cultivation” suggest ongoing efforts to optimize the growth and harvesting processes of algae to maximize yield and efficiency. The frequent mention of “astaxanthin,” a powerful antioxidant, highlights its importance in both food and nutraceutical applications. Overall, this trend analysis underscores the dynamic and evolving nature of research in this field, with a clear emphasis on health benefits, production optimization, and the exploration of specific bioactive compounds.

### Conceptual structure

**Fig. 10:**
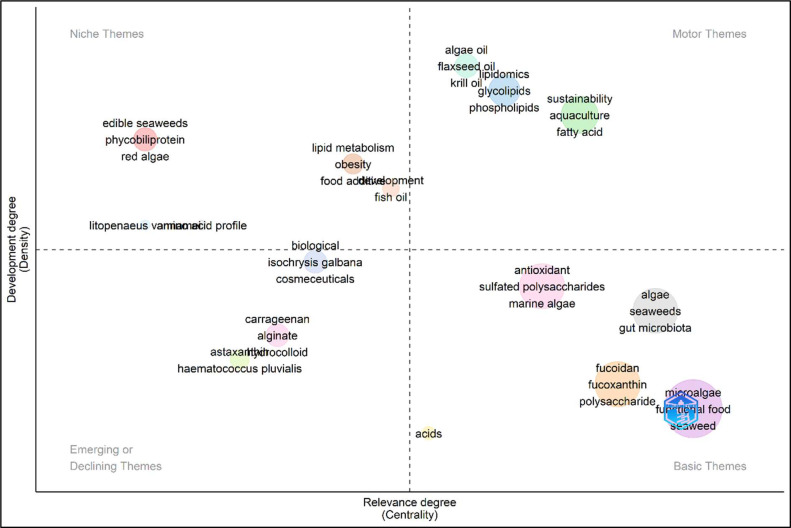
Algae utilization in foods research thematic/strategic map.

The thematic map (Fig. 10) results yield several key insights. Motor Themes (high centrality, high development) encompass algal extracts, which appear as a highly central and well-developed thematic cluster, underscoring their pivotal role and relative maturity in the research field. Bioactive compounds likewise constitute a central and advanced theme, reflecting their critical importance and extensive scholarly attention. In addition, polysaccharides, proteins, and lipids emerge as essential and thoroughly investigated topics, emphasizing their relevance for food science and technology applications. Niche Themes (high centrality, low development) include algae oil, which is conceptually central yet comparatively underdeveloped, thereby indicating substantial potential for further investigation and technological exploitation. Microalgae and seaweed, although central to the field, are still in stages of progressive development, suggesting ongoing research activity and increasing scientific and industrial interest. Basic Themes (low centrality, high development) comprise functional foods and nutraceuticals, which are well-developed but occupy a less central position in the thematic network. This pattern suggests that they function as foundational or supporting frameworks for more central, specialized themes. Similarly, antioxidant and anti-inflammatory properties represent well-established lines of inquiry that, while not thematically central, play a supportive role in the broader research context by underpinning studies on health-promoting effects. Emerging or Declining Themes (low centrality, low development) involve specific compounds such as fucoidan and carrageenan, which appear as less central and less developed. Their positioning indicates that they may represent either emerging research fronts with growing potential or areas experiencing a relative decline in scholarly focus.

**Fig. 11:**
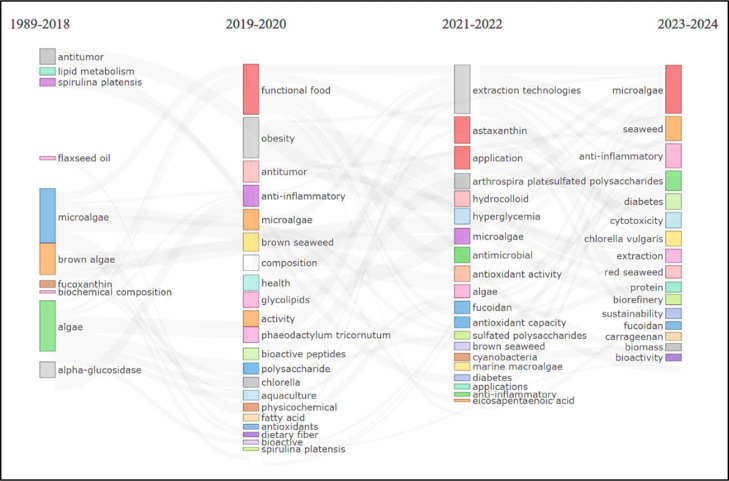
The thematic Sankey diagram illustrates research on the utilization of algae in food applications.

The thematic evaluation of the current state of research (Fig. 11) reveals several key trends over the period from 1989 to 2024. Early investigations (1989–2018) were predominantly oriented toward fundamental biochemical characterization and associated health effects, with a particular emphasis on lipid metabolism, biochemical composition, and specific algal species such as *Spirulina platensis*. Within this phase, research concentrated on topics including antitumor activities, polysaccharides, and phycocyanin. In the subsequent period (2019–2020), emerging trends reflected a transition toward targeted applications and health-promoting functionalities. Studies increasingly addressed functional foods, antioxidant and anti-inflammatory properties, and the potential role of algal-derived compounds, including astaxanthin and fucoxanthin, in the management of obesity and diabetes. More recent work (2021–2022) further broadened the research landscape by incorporating advances in extraction technologies and examining the application of microalgae in diverse health-related contexts. During this phase, there was a marked rise in interest in antimicrobial activity, hyperglycemia management, and cytotoxic effects. The most recent period (2023–2024) is characterized by a strong focus on the development of functional foods and nutraceuticals with clearly defined health benefits, particularly anti-inflammatory, antioxidant, and immunomodulatory effects. Concurrently, research attention has increasingly converged on specific bioactive compounds, including sulfated polysaccharides, fucoidan, carrageenan, and bioactive peptides.

**Fig. 12:**
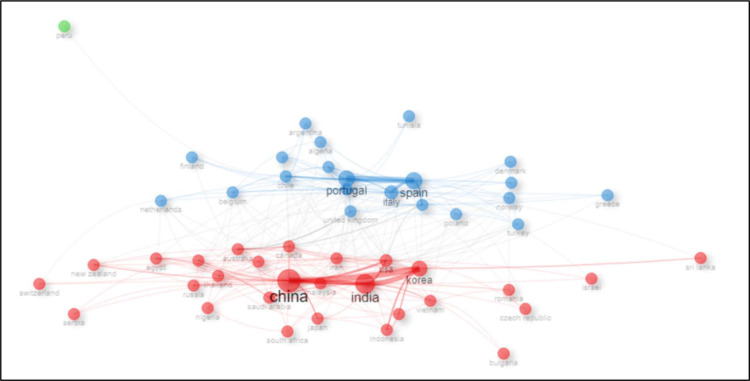
The analysis of the international collaboration network among countries depicts the global research landscape concerning the use of algae in food-related applications.

The collaboration network analysis of countries (Fig. 12) reveals that China and India occupy central positions within the network, underscoring their pivotal roles in international research collaborations. Additional countries, including Korea, Spain, Portugal, and Italy, also demonstrate substantial connectivity, maintaining multiple collaborative links with diverse partners. The network is organized into distinct clusters that predominantly reflect geographical and regional collaboration patterns. One such cluster comprises European countries such as Spain, Portugal, and Italy, while another encompasses Asian countries including China, India, and Korea. The edges connecting these nodes represent collaborative relationships and indicate that research topics such as bioactive compounds and polysaccharides are frequently co-investigated. This high degree of interconnectedness suggests that foundational research conducted in certain countries exerts a significant influence on subsequent studies elsewhere, thereby creating a dense web of international collaborations that collectively advance the field.

## Discussion

### Overview of algae utilization in foods

**Fig. 13:**
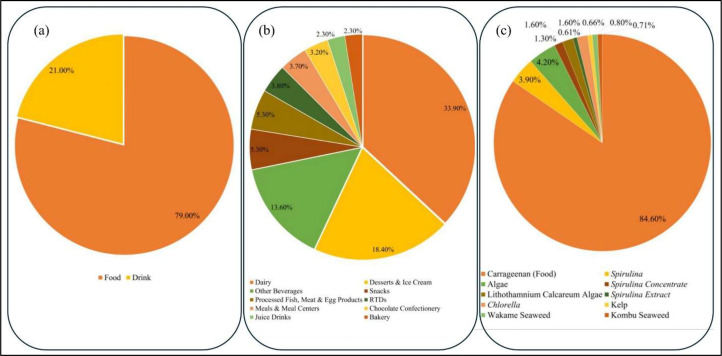
Shows the global market dependence on algae as ingredients in food and beverages (a) category kind (b) food category (c) algal ingredients [22].

As illustrated and examined by Boukid et al., [22] study that, in the period of 2015–2019, 13,090 new products with algal ingredients were introduced into the world market. The dataset (Fig. 13) shows that there is significant dominance in food applications, as 79 % of all launches consist of food products, and the other 21 % of all launches consist of beverages. This massive disequilibrium signifies that food-related industries are the major commercial powers in the algal products being taken into consideration in this paper. Whereas in the food industry, launches of products are highly marketed in a few segments. The largest percentage (33.90 %) of all food launches is comprised of dairy products, which indicates the high use of such ingredients in dairy products. Desserts and ice cream represent another 18.40 %, and along with dairy products, represent over half of the total food applications. Other beverages, snacks, processed fish, meat and egg products, ready-to-drink items, meals and meal centers, chocolate confectionery, juice drinks, and bakery products are also notable (5.30 %, 3.20 %, 2.30 % respectively) segments. The high concentration in dairy and dessert categories indicates that the ingredients are specifically desirable in their use in texture modification, stabilization, and nutritional value, and in formulations where specific rheological and sensory properties are necessary. Depending on that, the algal ingredients in these products are also skewed in the same way. Carrageenan is the most heavily used ingredient (84.60 % of all applications of algal ingredients) and thus confirms the long-time status of the substance as the main algal-based hydrocolloid in the food industry. Some secondary and minor ingredients are *Spirulina* (3.90 %), non-specified algae ingredients (4.20 %), *Spirulina* concentrate (1.30 %), *Lithothamnium calcareum* (1.60 %), and *Spirulina* extract (0.61 %). Other algal products, including *Chlorella*, kelp, wakame seaweed, and kombu seaweed, each occupy <1 % market share. On the whole, these other constituents comprise nearly 15.4 % of the use of algal ingredients, of which spirulina-based products constitute approximately 5.2 %. Such a pattern of distribution leads to the suggestion of an up-to-date, though still rather small-scale, diversification of algal applications beyond carrageenan, which could point to an increased, but still immature interest in the use of alternatives to carrageenan among food and beverage products.

### Key insights from the analysis

The bibliometric analysis indicates that research on algae as food additives, nutraceuticals, and sources of bioactive compounds has evolved from a relatively marginal topic into a rapidly expanding scientific domain. Temporal dynamics reveal a distinct inflection point around 2007, marking a transition from predominantly exploratory studies to a phase of accelerated, systematic investigation. This trajectory aligns with Price’s model of the development of scientific fields, which posits an initial exponential growth phase before eventual stabilization or maturation. The sustained annual publication growth rate of 13.42 % between 1989 and 2024 substantially exceeds the approximate 5.08 % growth rate observed across the scientific literature as a whole, demonstrating that research on algae in food-related applications is expanding at a markedly higher pace than the broader scientific enterprise. This pronounced acceleration reflects the growing recognition of algae as a strategically important resource for addressing global challenges related to food security, sustainable nutrition, and the development of novel pharmaceutical agents [23,24]. ​Publication output peaked in 2022 with 141 articles, followed by a moderate decline in 2023 and 2024 (116 and 82 articles, respectively). Rather than signifying a diminution of research interest, the observed pattern more plausibly reflects the intrinsic variability of annual publication counts in developing research domains, in conjunction with possible realignments of research funding priorities and cyclical shifts in thematic focus. The sustained upward trajectory through 2024, despite the reduction relative to the peak year, indicates continued dynamism and consolidation of the field. Moreover, these fluctuations align with patterns documented in other emerging research areas as they progress from predominantly foundational investigations toward phases oriented to translational and applied outcomes. The field thus appears to be evolving from broad exploratory inquiry to more targeted research on practical applications and commercial potential, as evidenced by the increasing focus on specific bioactive compounds and the development of functional foods.

Authorship patterns analysis shows that there is a high degree of collaborative research work, and the average number of co-authors is 6.02 per publication, and international co-authorship is 30.25 % of the publications. Such a level of cooperation is significantly higher than that indicated in other scientific sectors and indicates that algae studies are integrated within a very complex international research environment. The contribution of 4709 authors to 929 publications is an indication of the scope of the research community that has participated in this subject and includes representatives of various fields and geographical locations. Such influential members for production have become important actors providing consistent and stable research paths over several years. This is evidenced by their steady volume of publication, which is a commitment to the long-term principles of developing algae-based applications in the food industry. At the same time, the fact that the emerging researchers such as Lomartire Silvia and Hayes Maria are becoming more productive indicates active recruitment and efficient mentoring in the field, which is critical in sustaining and growing the research capacity in the long run. At an institutional level, it is found that the publication scene is dominated by European and Asian universities and research centers. The major centers of research activity are the University of Aveiro in Portugal (132 publications), Jeju National University in South Korea (84 publications), and the University of Porto in Portugal (66 publications). This institutional dispensation shows that Portugal has had adequate investment in the development of marine biotechnology and functional foods, accompanied by the strategic orientation of marine biotechnology and seaweed exploitation in South Korea. The strong influence of Asian organizations, especially in China and South Korea, may be explained by their geographical position near the rest of the marine resources and awareness of the economic and nutritional value of algae. The fact that contributing institutions have been spread all over the world, including Europe, Asia, Australia, the Americas, and the Middle East, highlights the worldwide recognition of the potential of this research field and a general desire to develop it further. The international collaboration network analysis indicates that China and India are the hubs of the global co-authorship network, and the other high levels of connections are with Korea, Spain, Portugal, and Italy. Such a set-up is indicative of the regional strongholds in marine science and the biased institutional investments in functional foods and associated aqua biotechnologies. The European institutional (Spain, Portugal, Italy) and Asian institutional (China, India, Korea) clustering implies that co-working relations are often pre-determined by the geographic and cultural proximity, but the fact that the interaction between these clusters is densely interconnected also means that cross-regional collaboration and knowledge sharing are high.

The strategic thematic map displays clear classes of research activity that are distinguished by a different degree of centrality and development. Whereas Motor Themes are highly centralized and well-developed and include algal extracts, bioactive compounds, polysaccharides, proteins, and lipids. These developed themes have shown conceptual significance in the field as well as notable scholarly maturity, which shows well-developed research areas that can be applied in development and commercialization. These topics have been categorized as motor themes, indicating that the research society has reached a considerable agreement on the significance and methodological strategies of studying these elements. While Niche Themes, such as algae oil and microalgae-based compounds, are in the middle of the research network but are relatively unexplored, this means that innovative research and technological growth have a high potential. The positioning implies the existence of conceptual recognition in these areas, but without vast empirical research and technological implementation opportunities. The niche positioning of algae oil is especially important because the commercial interest in omega-3 fatty acids derived from algae sources is growing as an alternative source to fish oil, and thus, researchers can consider the gaps in knowledge and technological constraints. To support the analysis of the thematic evolution, shows various phases of research with respect to changing priorities in research. Initial studies (1989–2018) focused on basic biochemical characterization and species-specific attributes, with specific attention being given to lipid metabolism and phycobiliproteins. The shift in the transitional period (2019–2020) was associated with the orientations on targeted functional applications, and research began to focus more on antioxidant and anti-inflammatory effects and the possible therapeutic use of metabolic diseases. Recent efforts (2021–2022) broadened the scope of investigation to include antimicrobial potential and extraction technology innovations and the current period (2023–2024) is defined by the focused attention on the creation of functional foods and nutraceuticals with precisely defined health claims, especially on anti-inflammatory, antioxidant, and immunomodulatory effects and exploration of the potential of certain bioactive compounds such as fucoidan, carrageenan, and bioactive peptides. This developmental process of specific research stages can be considered as the maturity line of the model introduced by Price, when the field starts with the exploratory level of investigation and then passes to the stage of development of practical application. The change of species to individual compound research and application in particular health care areas may indicate that the field is no longer focused on knowledge and more application-oriented, which will place the research community in a position to achieve clinical translation and commercialization of events in the future.

## Conclusions

The current bibliometric analysis of 929 publications (1989–2024) demonstrates that research on algae as food additives and nutraceutical ingredients has progressed from a marginal topic to a dynamic and globally networked field of inquiry. Exhibiting an annual growth rate of 13.42 % and a marked inflection point around 2007, this domain now positions algae as a strategic resource for food security, sustainable nutrition, and pharmaceutical innovation. The field is characterized by substantial international collaboration, with 30.25 % of studies involving co-authorship across countries, and pronounced interdisciplinary engagement. European and Asian institutions, particularly those in Portugal, South Korea, and China, emerge as key contributors to knowledge production. Research trajectories have evolved from foundational biochemical characterization toward application-oriented studies, with recent investigations focusing on functional foods and specific bioactive compounds, including fucoidan, carrageenan, and bioactive peptides with documented health-related effects. Core thematic areas such as algal polysaccharides, proteins, and lipids exhibit both scientific maturity and notable commercial relevance, while emerging topics, especially microalgae-derived compounds, indicate expanding research frontiers and innovation potential. Current commercial applications are predominantly concentrated in dairy and dessert matrices, where carrageenan plays a central role. However, the increasing use of spirulina and other algal bioactives points to a gradual reorientation toward functional foods bearing explicit health claims. Priority directions for future research include: (i) rigorous in vivo validation of biological activities; (ii) development and optimization of cost-effective and scalable extraction and purification technologies; (iii) systematic exploration and characterization of novel algal species and strains; (iv) establishment of harmonized quality, safety, and standardization protocols; and (v) implementation of environmentally and economically sustainable production systems. Collectively, these trends indicate that the field is undergoing a critical transition from predominantly knowledge-generating research to translational and industrial application, with substantial potential to underpin sustainable, health-oriented food systems.

## Declaration of competing interest

The authors have no declared conflicts of interest.

## Data Availability

Data will be made available on request.
